# Gambling with Your Health: Associations Between Gambling Problem Severity and Health Risk Behaviours, Health and Wellbeing

**DOI:** 10.1007/s10899-019-09902-8

**Published:** 2019-11-08

**Authors:** Nadia Butler, Zara Quigg, Rebecca Bates, Madeleine Sayle, Henrietta Ewart

**Affiliations:** 1 Public Health Institute, Liverpool John Moores University, 3rd Floor Exchange Station, Tithebarn Street, Liverpool, L2 2QP UK; 2 Department of Health and Social Care, Public Health Directorate, Douglas, Isle of Man

**Keywords:** Problem gambling, Health, Smoking, Alcohol, Diet, Exercise

## Abstract

The current study examined the association between gambling problem severity and health risk behaviours, health and wellbeing. A cross-sectional survey (including representative population and supplementary convenience samples) was conducted with 2303 adult residents of a British Island. Gambling problem severity was assessed using the Problem Gambling Severity Index. The EQ-5D-5L, WEMWBS and AUDIT-C were used to measure general health, mental wellbeing and alcohol use, respectively. Other measures included diet, physical exercise and tobacco use. Differences between gambling severity levels for each measure were analysed using logistic regression adjusting for age, sex and income. Compared to non-problem gamblers, moderate/high severity gamblers had higher odds of a poor diet, low physical exercise and poor general health. Tobacco use was associated with both low and moderate/high severity gambling. Low severity, but not moderate/high severity gambling, was significantly associated with binge and higher risk drinking behaviours. Health risk behaviours tended to cluster, with a graded relationship between gambling problem severity and odds of reporting at least two health risk behaviours. Compared to non-problem gamblers, low severity gamblers were approximately twice as likely and moderate/high severity gamblers were three times as likely, to have low mental wellbeing. Findings suggest associations between gambling problems and a range of health risk behaviours and health issues, and crucially that such issues are not limited to gamblers with the highest severity of problems. Addressing gambling across the whole continuum of risk should be a key public health priority.

## Introduction

Globally, there has been increasing calls to recognise gambling as a public health issue (Korn and Shaffer 1999; British Medical Association 2007; Wardle et al. 2012; Marshall 2009). Such calls have been driven in part by emerging findings linking gambling to other key public health concerns (Okunna et al. 2016). Recreational gambling has been shown in several studies to be significantly associated with alcohol abuse, smoking, and illicit drug use (Okunna et al. 2016; Liu et al. 2009; Desai et al. 2006). Recreational gamblers are also more likely to report poor physical and mental health compared to non-gamblers (Okunna et al. 2016; Morasco et al. 2006).

While research has found that engagement in recreational gambling has significant correlations with health and health risk behaviours, problem or pathological gamblers may be at increased risk of adverse health and behavioural issues (Subramaniam et al. 2015). Pathological gambling is defined as a distinct disorder in the Diagnostic and Statistical Manual of Mental Disorders (DSM-V) and has been found to be highly comorbid with other DSM-V disorders (American Psychological Association 2013), including mood, anxiety, attention-deficit and personality disorders (Petry et al. 2005; el-Guebaly, et al. 2006; Lorains et al. 2011; Specker et al. 1995). A national survey in the US found that 96.3% of respondents with lifetime pathological gambling also met the criteria for another DSM-IV disorder (Kessler, et al. 2008). Further, studies show high prevalence estimates for nicotine dependence, alcohol use disorder, illicit drug abuse and substance abuse disorders amongst pathological gamblers (Lorains et al. 2011). Such findings may be unsurprising, considering gambling disorder is defined in the DSM-V under the substance-related and addictive disorders criteria, reflecting evidence that gambling behaviours activate similar reward systems to drugs and produces behaviours comparable to those associated with substance use disorders (American Psychological Association 2013). There is also evidence to suggest that similar genetic, environmental and social factors may influence the co-development and maintenance of substance-related and addictive disorders (Potenza 2006).

Studies to date have been limited for the most part by making comparisons using a diagnostic perspective which takes a dichotomous approach using DSM-V cut-offs to define pathological gambling (Orford et al. 2010). However, as with other public health issues such as alcohol use, gambling is associated with a continuum of risk, where pathological gambling represents the extreme (el-Guebaly, et al. 2006). A public health approach would encompass a broader perspective to gambling across the continuum of risk and not be limited to a narrow focus on pathological gambling (Shaffer and Korn 2002). Wardle and colleagues argue that gamblers who are at-risk of developing gambling problems are of equal concern, as they represent not only a larger ‘vulnerable’ group who may experience problems, but also a group whose problems may worsen over time (Wardle et al. 2012). Further, public health policies aimed at addressing the negative associated effects of gambling will, for the most part, be aimed at this sub-clinical group, including those who never go on to develop clinical symptoms of problem gambling. A study of physical and mental health of gamblers in England created arbitrary cut-offs of DSM criteria to categorise at-risk and problem gamblers. While some associations were found between alcohol use, smoking and anxiety disorders and at-risk gambling, interpretation of the findings may have been limited by the imperfect operationalisations of the continuum of gambling severity (Cowlishaw and Kessler 2016). Use of the DSM in this way, as a screening measure of problem gambling in general populations, is problematic as the DSM’s primary purpose is to diagnose a mental health disorder (i.e. gambling disorder) (Wynne 2004). While it has been shown to be generally successful in identifying the most serious problem gambling, it is arguably less appropriate for identifying individuals with sub-clinical symptoms.

Other measures have been developed which attempt to assess facets of problem gambling over a continuum of risk. The Problem Gambling Severity Index (PGSI), developed as part of the broader Canadian Problem Gambling Index (CPGI), is a quantitative scale summed to determine a total gambling severity score. PGSI scores are then typically categorised as four gradations of severity level from non-problem gambling to problem gambling. The PGSI has been demonstrated to show greater classification accuracy in identifying those at risk of developing gambling problems than the DSM or other population level measurement tools (Orford et al. 2010). Studies which use the PGSI to examine the association between levels of gambling severity and health have shown a higher prevalence of comorbid conditions including substance abuse and mood disorders amongst low-risk gamblers compared to non-problem gamblers, with the highest prevalence amongst moderate/high risk gamblers (el-Guebaly, et al. 2006). For example, 2% of non-problem gamblers in a Canadian population study were alcohol dependent compared with 7% of low-risk and 15% of problem gamblers (Marshall and Wynne 2004). This suggests that there may be a correlation between gambling severity and increased vulnerability to other health issues and health risk behaviours.

Through the use of the PGSI to distinguish low, moderate and high severity gamblers, the current study aims to determine whether there is an association between gambling problem severity and: (1) health risk behaviours including alcohol consumption, tobacco use, poor diet and low physical activity; and, (2) general health and mental wellbeing.

## Methods

The sample was drawn from a British Island with a population of 67,100 adults aged 18+ years (79.1% of all residents). The mean age of all residents on the island was 42.5 and 42.1% of the population were aged 55+ years. The British Island has a variety of on-island gambling environments in which residents can participate in gambling activities, including nine betting offices and fixed odds betting terminals, three adult gaming centres, a bingo hall and a casino, in addition to 96 pubs, bars and clubs which have fruit or slot machines (Isle of Man Government 2019a). Residents are able to purchase tickets for both the National Lottery Draw and the Irish Lottery Draw, and over forty online gambling organisations hold licenses for online gambling websites (Isle of Man Government 2019b).

### Study Design and Participants

A cross-sectional survey of residents aged 18+ years was undertaken in October 2017. The survey was conducted in two phases. Phase 1 was an invited representative population sample. Based on a response rate of 25% for a previous similar survey (The Centre for Public Innovation 2018), 7000 addresses were selected, split by postcode areas, and proportionally selected based on census data of population numbers. Random sampling was used to select addresses within each postcode, and respondents within each household were selected using the next birthday method. The number of completed surveys from this phase was n = 1131; with a response rate of 16.2% which represented 1.7% of the adult population. Following completion of phase 1, phase 2 was implemented. It consisted of a convenience sample, promoted as open access to the general public through media channels, and received 1172 responses. The total sample size was N = 2303 (3.4% of the adult population).

### Measures

#### Problem Gambling Severity Index (PGSI)

The PGSI is a self-reported instrument, consisting of nine items, used to measure severity of gambling problems in general populations (Ferris and Wynee 2001; Holtgraves 2008). Four items measure difficulties in controlling gambling (chasing loses, escalating gambling to maintain excitement, betting more money than you can afford to lose, borrowing money for gambling) and five items assess adverse consequences of gambling (awareness of gambling problem, health problems, financial difficulties, feelings of guilt, and criticism of gambling behaviour by others). The PGSI items are measured on a four point scale (*0 *= *never, 1 *= *occasionally, 2 *= *fairly often, 3 *= *very often*). Total scores (range 0–27) were calculated and classified into four gambling severity categories: non-problem (0); low severity (1–2); moderate severity (3–7); and high severity (8+). Due to sample sizes and in line with previous categorisation of severity, the moderate and high severity gambling categories were amalgamated (el-Guebaly, et al. 2006).

#### Health Risk Behaviours and Health and Wellbeing Measures

Seven measures of health risk behaviours and health and wellbeing were included in the analysis: smoking, binge drinking, higher risk drinking, poor diet, low physical activity, low mental wellbeing and poor general health. Smoking was defined as current smoking of tobacco on a daily basis. Alcohol consumption was measured using AUDIT-C questions (Bush et al. 1998). Higher risk drinking was defined as a score of > 5 on the AUDIT-C. Binge drinking was defined as ≥ 6 drinks (i.e. one standard UK unit of alcohol, equivalent to 10 ml pure alcohol) per occasion ≥ 1 day a week. Poor diet was defined as eating < 2 portions of fruit and vegetables (excluding potatoes) a day. Low physical activity was defined as taking part in < 150 min of physical activity (e.g. walking quickly, cycling, sports or exercise) in the past week. Mental wellbeing was measured using the Warwick-Edinburgh Mental Wellbeing Scale (WEMWBS) (Stewart-Brown, et al. 2009). Low mental wellbeing was defined as scores of ≤ 40 based on previous research (The Centre for Public Innovation 2018). The EQ-VAS (part of the EQ-5D-5L instrument) is a self-reported measure of general health recorded on a vertical visual scale (range 0–100), where the end points represent ‘the best health you can imagine’ and ‘the worst health you can imagine’ (van Reenen and Janssen 2015). Scores were dichotomised to indicate poor general health as > 1 standard deviation (17.5) below the mean (79.3), thus poor general health was operationalised as scores < 62.

#### Sociodemographic Factors

Sociodemographic factors included: sex, age (18–34; 35–54; 55+ years), and income level (*rounded to the nearest 1000)* (< £20,000; £20,000–£79,000; £80,000+).

### Analysis

Analyses were undertaken in SPSS (v.25). Bivariate analyses were used to highlight the association between gambling severity categories and sociodemographics (Table 1). χ^2^ tests were also used to explore unadjusted associations between gambling severity categories and health risk behaviours, and general health and mental wellbeing. Binary logistic regression (backward likelihood ratio) models were used to estimate the association between gambling severity categories and each measure of interest after controlling for sociodemographics. All models were also ran including the PGSI total score as an independent continuous variable rather than the PGSI severity threshold categories. This was to determine whether the gambling severity thresholds are useful in exploring relationships with health risk behaviours and health and wellbeing, and to ensure that our amalgamation of the moderate/high severity categories does not obscure relationships (Harrison et al. 2016). Logistic regressions for each dependent measure were first ran with PGSI thresholds as an independent categorical variable, and then reran with PGSI total score as an independent continuous variable. To explore the potentially mediating effect of health risk behaviours on the association between gambling severity and general health and mental wellbeing, regressions using gambling severity as a continuous variable were reran with health risk behaviours included in the models.

**Table 1:** Sample characteristics by PGSI category

	All		PGSI category^a^				
	N	%	Non-problem, % (n)	Low severity, % (n)	Moderate/high severity, % (n)	χ^2^	*p*
Prevalence			93.2 (1666)	5.0 (89)	1.8 (33)		
Sex							
Male	833	38.2	91.2 (640)	6.4 (45)	2.4 (17)		
Female	1345	61.8	94.5 (1018)	4.1 (44)	1.4 (15)	7.605	0.022
Age (years)							
18–34	283	13.1	83.4 (191)	12.7 (29)	3.9 (9)		
35–54	837	38.8	92.1 (630)	5.8 (40)	2.0 (14)		
≥ 55	1037	48.1	96.7 (825)	2.3 (20)	0.9 (8)	52.918	< 0.001
Income level (£)							
< 20,000	271	16.3	91.5 (238)	5.8 (15)	2.7 (7)		
20,000–79,000	1138	68.6	92.5 (1015)	5.8 (64)	1.6 (18)		
> 80,000	251	15.1	93.9 (230)	3.7 (9)	2.4 (6)	3.416	0.491

^a^PGSI category scores: non-problem (0); low severity (1–2); moderate/high severity (3+)

## Results

### Prevalence of Gambling Severity and Association with Demographics

Three quarters (74.5%; n = 1406) of study participants reported gambling at least once in the past 12 months. The majority (93.2%; n = 1666) of participants were non-problem gamblers, while 5.0% (n = 89) fell in the low severity category of the PGSI and 1.8% (n = 33) fell in the moderate/high severity category. Table 1 shows demographic characteristics, health risk behaviours, and health outcomes across PGSI severity categories. According to χ^2^ tests there was a higher prevalence of low and moderate/high severity gamblers amongst those aged 18–34 years compared to those aged 35–54 years or 55+ years (*p* < 0.001) and amongst males compared to females (*p* = 0.022). There was no significant association between gambling severity and income level.

### Health Risk Behaviours Associated with Gambling Severity

In bivariate analyses the prevalence of all health risk behaviours except binge drinking was highest amongst moderate/high severity gamblers and lowest amongst non-problem gamblers (Table 2). The prevalence of binge drinking was highest amongst low severity gamblers. Further, there was a significant graded relationship between gambling severity and the prevalence of ≥ 2 health risk behaviours, with 19.3% of low severity gamblers and 31.3% of moderate/high severity gamblers having at least two health risk behaviours.

**Table 2:** Health risk behaviours, general health and mental wellbeing, by PGSI category

Measure	All		PGSI category^a^				
	N	%	Non-problem, % (n)	Low severity, % (n)	Moderate/high severity, % (n)	χ^2^	*p*
Smoking (daily)	1965	8.2	7.5 (125)	16.9 (15)	24.2 (8)	20.750	< 0.001
Binge drinking	1744	21.1	20.1 (301)	38.3 (31)	34.5 (10)	18.356	< 0.001
Higher risk drinking	1525	31.5	30.7 (405)	45.1 (32)	48.0 (12)	9.494	0.009
Poor diet	2099	6.7	6.4 (107)	11.2 (10)	27.3 (9)	23.808	< 0.001
Low physical activity	1988	18.6	18.0 (294)	12.6 (11)	39.4 (13)	11.812	0.003
≥ 2 Health risk behaviours^b^	1878	9.2	8.6 (137)	19.3 (17)	31.3 (10)	28.906	< 0.001
Low mental wellbeing	1817	16.7	15.2 (242)	29.2 (26)	46.7 (14)	32.057	< 0.001
Poor general health	1888	14.2	12.8 (211)	17.0 (15)	34.4 (11)	13.641	0.001

^a^PGSI category scores: non-problem (0); low severity (1–2); moderate/high severity (3+)

^b^Includes smoking (daily), binge drinking, poor diet and low physical exercise

In multivariate analysis, after adjusting for age, sex and income, associations between all health risk behaviours and gambling severity categories remained significant. Moderate/high severity gamblers were four times more likely to have a poor diet than non-problem gamblers and 2.9 times more likely to have low physical activity (Table 3). Low severity gamblers were twice as likely to smoke daily compared to non-problem gamblers, whilst moderate/high severity gamblers were over three times more likely to be smokers. Low severity gamblers were approximately twice as likely to be binge or higher risk drinkers than non-problem gamblers. Moderate/high severity gambling was not significantly associated with binge or high risk drinking. Finally, low severity gamblers were 2.2 times more likely, and moderate/high risk gamblers were 4.6 times more likely, to have ≥ 2 health risk behaviours than non-problem gamblers.

**Table 3:** Independent relationships between PGSI categories and health risk behaviours, general health and mental wellbeing

Measure	N	*p*	PGSI category (reference category non-problem)^a^			
			Low severity		Moderate/high severity	
			AOR (95% CI)	*p*	AOR (95% CI)	*p*
Smoking (daily)	1571	0.004	2.063 (1.119–3.802)	0.020	3.272 (1.324–8.085)	0.010
Binge drinking	1425	< 0.001	2.686 (1.641–4.395)	< 0.001	1.864 (0.775–4.482)	0.164
Higher risk drinking	1240	0.004	2.078 (1.245–3.469)	0.005	2.371 (0.985–5.704)	0.054
Poor diet	1573	0.008	1.482 (0.727–3.021)	0.279	4.000 (1.615–9.910)	0.003
Low physical activity	1553	0.012	0.695 (0.363–1.331)	0.272	2.911 (1.349–6.284)	0.006
≥ 2 Health risk behaviours^b^	1520	< 0.001	2.217 (1.237–3.971)	0.007	4.595 (1.985–10.636)	< 0.001
Low mental wellbeing	1516	0.002	1.713 (1.034–2.837)	0.037	3.351 (1.477–7.600)	0.004
Poor general health	1564	0.003	1.411 (0.785–2.536)	0.249	3.952 (1.754–8.903)	0.001

Adjusted for age, sex, income level

*AOR* adjusted odds ratio, *CI* confidence interval

^a^PGSI category scores: non-problem (0); low severity (1–2); moderate/high severity (3+)

^b^Includes smoking (daily), binge drinking, poor diet and low physical exercise

Logistic regressions were then rerun with PGSI score as an independent continuous variable. After adjusting for sociodemographics, associations between smoking and poor diet and PGSI gambling severity score remained significant. An increase in PGSI score by one increased the odds of: smoking by 16% (AOR = 1.162; CIs = 1.062–1.271) and poor diet by 13% (AOR = 1.131; CIs = 1.033–1.240). PGSI severity score was not significantly associated with low physical activity, binge or higher risk drinking. The odds of experiencing ≥ 2 health risk behaviours were 13% higher with an increase in PGSI score by one (AOR = 1.131; CIs = 1.030–1.242).

### Associations Between Gambling Severity and General Health and Mental Wellbeing

In bivariate analysis the prevalence of both low mental wellbeing and poor general health was highest amongst moderate/high severity gamblers (Table 2). In multivariate analysis, after adjusting for age, sex and income, findings showed that, relative to non-problem gamblers, moderate/high severity gamblers were approximately four times more likely to have poor general health (Table 3). Low severity gamblers were not significantly more likely to have poor general health than non-problem gamblers. Compared to non-problem gamblers, low severity gamblers were 1.7 times more likely, and moderate/high severity gamblers were 3.4 times more likely, to have low mental wellbeing (Table 3).

After rerunning logistic regressions with PGSI score as a continuous variable, associations between both measures and PGSI gambling score severity remained significant. An increase in PGSI score by one increased the odds of: poor general health by 15% (AOR = 1.152; CIs = 1.052–1.262) and low mental wellbeing by 17% (AOR = 1.166; CIs = 1.054–1.289). Analysis demonstrated that even when all health risk behaviours were added to the model, PGSI gambling severity score remained significantly associated with low mental wellbeing (AOR = 1.134; CIs = 1.009–1.274). PGSI score was no longer significantly associated with poor general health, after adding all health risk behaviours to the model (AOR = 1.124; CIs = 0.998–1.265).

## Discussion

Findings from the present study are in line with previous research demonstrating the association between problem gambling and several health risk behaviours. Crucially, our study demonstrated that health risk behaviours amongst at-risk and problem gamblers tended to cluster, with a graded relationship between gambling severity and the odds of reporting at least two health risk behaviours, including smoking, binge drinking, poor diet and low physical exercise. Problem gamblers, therefore, represent a cohort more vulnerable to other health risk behaviours compared to non-problem gamblers, and crucially, are more likely to experience more than one. Exposure to more than one health risk behaviour is significant as such risks interact to create a multiplicative increase in morbidity (Bellis et al. 2016). For instance, obesity (from poor diet and low physical exercise) and regular alcohol consumption interact to increase risks of liver disease mortality to a greater extent than each individual risk (Hart et al. 2010). Similarly, alcohol consumption and smoking interact and increase the risk of developing cancer (Pelucchi et al. 2006).

The current study found that low severity, but not moderate/high severity problem gambling, was significantly associated with binge and higher risk drinking behaviours. This suggests for low severity gamblers at least, the two behaviours may be linked. Opportunities and promotion of gambling are often associated with environments where the primary focus is alcohol consumption, for example gaming machines and gambling adverts during sports events televised in licensed premises. Conversely, alcohol is often a key feature of gambling focused environments such as racecourses. Such factors may shift gamblers along the continuum to higher levels of risk or problem severity (Hing et al. 2014). Alcohol intoxication is associated with increased motivations to gamble, more risky gambling behaviour and higher loses, whilst gambling advertising and promotions have been found to remind problem gamblers about gambling and arouse urges to gamble (Barrett et al. 2015; Binde 2009; Leino et al. 2017). For some individuals, higher losses may serve as a pathway to future gambling problems if they chase their losses (Lesieur 1984). Such environments may therefore represent a crucial setting for public health interventions focused on raising the awareness of gambling problems amongst lower risk gamblers. Harm reduction initiatives could include healthy gambling guidelines similar to low-risk drinking guidelines (Shaffer and Korn 2002). Such a focus on at-risk gamblers are likely where the greatest public health advances could be made, with the higher prevalence of such individuals producing a greater aggregate of associated problems than pathological gamblers (Shaffer and Korn 2002). Further, similar to problem drinking, at-risk gamblers may be more amenable to intervention than more severe problem gamblers (Shaffer and Korn 2002).

Our study also found associations between gambling severity and health and wellbeing. Exploratory analysis, which controlled for the mediating effects of health risk behaviours (i.e. poor diet, low exercise, tobacco use, alcohol consumption) on general health and mental wellbeing, showed that gambling severity remained associated with low mental wellbeing, but was no longer significantly associated with poor general health. This suggests that gambling severity has direct effects on mental wellbeing beyond its associations with health risk behaviours. It also suggests that gambling severity has an indirect effect on general health via association with health risk behaviours. Irrespective of the pathways between gambling problems and poor health, that this cohort has increased risk of poor health and wellbeing may have implications for gambling intervention opportunities. At present in the UK there is a lack of gambling-specific treatment, with a recent study of national service provision finding only one dedicated gambling treatment unit (Rigbye and Griffiths 2011). Furthermore, at-risk gamblers would likely fall below the threshold for accessing such specialised support. However, many of the other associated health behaviours (e.g. poor diet) and outcomes (e.g. poor general health) may present at generalist services, which could provide opportunities to identify and address at-risk or problem gambling behaviour. Previous research has demonstrated that at-risk and problem gamblers access such services at a higher rate than non-problem gamblers (Cowlishaw and Kessler 2016).

The findings in the current study should be interpreted in light of limitations. The cross-sectional nature of the study prevents an evaluation of causality and while associations were determined, it cannot be known whether gambling problems precede health risk behaviours and health issues or vice versa. Despite the relatively large sample size, at-risk and problem gambling had a relatively low prevalence. When sample sizes are small, sampling error can conceal study effects. Similar to practice elsewhere, we combined moderate and high severity gamblers (el-Guebaly et al. 2006). This may have obscured some patterns of association which could be made clearer in future research with larger sample sizes for each severity level. To mitigate the risk of this we also ran our analyses using the PGSI continuous total score, and whilst most associations remained significant, risky alcohol consumption did not. Our analyses using the categories of the PGSI demonstrated that low severity gamblers were associated with risky alcohol use measures and the use of the continuous PGSI total score obscures this effect. Our use of both a continuous and categorical approach to the PGSI is in line with previous studies and demonstrates the value of using both the continuous score and categorical thresholds of severity (Farrell 2018).

While there has been increasing calls in recent times to view gambling as a public health issue, it has not traditionally been considered within the discipline (Griffiths 2004; The Lancet 2017). Thus, the health and social consequences associated with gambling were poorly understood, particularly with respect to gamblers who fall below commonly used thresholds for problem gambling, such as those defined in a medical model (e.g. the DSM). Most research to date has been limited by utilising a diagnostic perspective which takes a dichotomous approach to problem gambling, thus the broader continuum of gambling risk is not considered. The current study’s use of a gambling measure, the PGSI, which can delineate at population level, problem gamblers from at-risk gamblers, is more useful in considering gambling and associated harms and strengthening the case for gambling to be considered within a public health framework. This study indicates associations between gambling severity and a number of health risk behaviours and health issues, and, crucially, that such issues are not confined to gamblers with the highest severity of problems. Current UK National Health Service guidance on gambling problems allows individuals to assess if they are a problem gambler by scoring eight or more on the PGSI (NHS 2017). By viewing gambling solely from a medical model with such defined thresholds, intervention efforts will target only a small proportion of those affected by gambling associated problems. The current study has demonstrated the value in considering gambling and associated health behaviours across the whole continuum of gambling problem severity and the potential public health implications of this.
